# The predictive value of glycated CD59 for fetal overgrowth in second-trimester pregnant women

**DOI:** 10.3389/fendo.2026.1924289

**Published:** 2026-10-01

**Authors:** Xiaofan Lu, Hui Zhou, Qiang Gang, Mengmeng Dou, Ying Zhang, Yu Sun

**Affiliations:** 1 Department of Endocrinology, Suqian Hospital Affiliated to Xuzhou Medical University, Suqian, China; 2 Department of Endocrinology, Suqian Clinical College of Xuzhou Medical University, Suqian, China; 3 Department of Endocrinology, Jiangsu Province Suqian Hospital, Suqian, China; 4 Department of Endocrinology, Nanjing Drum Tower Hospital Group Suqian Hospital, Suqian, China

**Keywords:** adverse pregnancy outcomes, biomarker, gestational diabetes mellitus, glycated CD59, large for gestational age, macrosomia

## Abstract

**Objective:**

To investigate the predictive value of glycated CD59 (gCD59) during mid-pregnancy for adverse pregnancy outcomes, specifically macrosomia and large for gestational age (LGA), in a prospective cohort of pregnant women undergoing oral glucose tolerance testing (OGTT), with adjustment for gestational diabetes mellitus (GDM) status.

**Methods:**

A total of 672 pregnant women who attended prenatal visits at the Affiliated Suqian Hospital of Xuzhou Medical University from January 2022 to July 2023 were enrolled, and baseline data were collected via the Zhendings Maternal and Child Health System. At 24–28 weeks of gestation, an oral glucose tolerance test (OGTT) was performed. Based on the results, participants were divided into a GDM group and a normal glucose tolerance (NGT) group for descriptive comparisons; for all predictive analyses, GDM status was included as a covariate in multivariable models. Concurrently, gCD59 levels were measured using the ELISA method. The pregnant women were followed up until after delivery, and data on delivery-related information and pregnancy outcomes were collected. Differences in pregnancy outcomes between the two groups were compared. Logistic regression analysis was used to identify risk factors for adverse pregnancy outcomes, and the predictive value of gCD59 levels for adverse pregnancy outcomes was assessed using ROC curves.

**Results:**

Macrosomia and Large for Gestational Age (LGA) were the two most common adverse pregnancy outcomes. Compared with the NGT group, the incidence of macrosomia and LGA was significantly higher in the GDM group (*P* < 0.05). Compared with the NGT group, gCD59 levels were significantly higher in the GDM group (1.49 (0.90, 2.33) vs. 0.87 (0.39, 1.62), P < 0.001). Logistic regression analysis indicated that gCD59 was an independent risk factor for macrosomia and LGA, and the incidence of macrosomia and LGA increased with rising gCD59 levels. In predictive modeling, the addition of gCD59 to traditional risk factors improved the AUC for macrosomia from 0.770 (95% CI: 0.679–0.833) to 0.864 (95% CI: 0.823–0.904) (DeLong *P* = 0.046); for LGA, the AUC improved from 0.723 (95% CI: 0.663–0.782) to 0.790 (95% CI: 0.737–0.843) (DeLong *P* = 0.103).

**Conclusion:**

Macrosomia and LGA were the most common adverse pregnancy outcomes in the study cohort. gCD59 was identified as an independent risk factor for both macrosomia and LGA. In predictive modeling, gCD59 demonstrated statistically significant incremental value for macrosomia (AUC: 0.770 vs. 0.864, DeLong *P* = 0.046), while for LGA the improvement showed a favorable trend but did not reach statistical significance (AUC: 0.723 vs. 0.790, DeLong *P* = 0.103). These findings suggest that gCD59 may serve as a supplementary biomarker, particularly for risk stratification of macrosomia, with potential utility for identifying pregnancies at risk of excessive fetal growth.

## 1 Introduction

Gestational diabetes mellitus (GDM), a common comorbidity of pregnancy, brings short-term and long-term adverse outcomes for both mothers and infants (1). Adverse pregnancy outcomes can be affected by a variety of maternal, placental, and fetal factors. Identification of risk factors and early stratification of high-risk pregnant women are of great clinical significance to protect maternal and fetal health (2).

Recently, there has been a notable advancement in the field of GDM research, with the identification of novel biomarkers for the precise diagnosis and treatment of the condition (3). Glycated CD59 (gCD59) has shown an encouraging performance in diagnosing GDM. CD59, an inhibitor of membrane attack complex (MAC) widely distributed on the cell membrane, protects host cells against complement-mediated damage (4). Shed from the cell membrane by phospholipases, gCD59 is soluble and stably expressed in blood and urine, showing a significant association with blood glucose levels (5). Evidence has pinpointed a close link between gCD59 level and hyperglycemia during pregnancy (6). Our previous study has consistently demonstrated that gCD59 is a potential diagnostic marker of GDM (7). As shown by literature review, the potential of gCD59 in predicting adverse pregnancy outcomes, especially in the Chinese population of GDM, has been sporadically analyzed (8). In this prospective cohort study, we aimed to assess the capability of gCD59 in predicting adverse pregnancy outcomes, specifically macrosomia and LGA, in a cohort of Chinese pregnant women undergoing routine OGTT screening, with adjustment for GDM status. By including both women with GDM and those with normal glucose tolerance, we sought to evaluate the incremental predictive value of gCD59 beyond conventional risk factors in the general obstetric population.

## 2 Methods

### 2.1 Study population

This was a prospective cohort study involving pregnant women who for the first time visited the Obstetrics Clinic of the Affiliated Suqian Hospital of Xuzhou Medical University from January 2022 to July 2023. The flowchart of participant allocation is shown in **Figure 1**. The study population included pregnant individuals between the ages of 20 and 40 years and with gestational ages ranging from 24 to 28 weeks, with singleton intrauterine pregnancies confirmed through color Doppler ultrasound. Women who were pregnant and had a history of type 2 diabetes mellitus (T2DM), severe organic or primary diseases (e.g. liver and kidney dysfunction, heart failure, cerebral infarction, and malignancies), and cognitive/affective/motor/communication disorders were excluded from the study. For the purposes of the predictive analyses, the entire cohort (including both GDM and NGT participants) was used, with GDM status (yes/no) included as a covariate in all multivariable regression models to adjust for its potential confounding effect. This approach was chosen to preserve statistical power and to evaluate the performance of gCD59 in the general pregnant population undergoing routine screening. Written informed consent was provided by all included pregnant women. An approval for a prospective cohort study was obtained from the Ethics Committee of the Affiliated Suqian Hospital of Xuzhou Medical University (No. 2021011).

**Figure 1:**
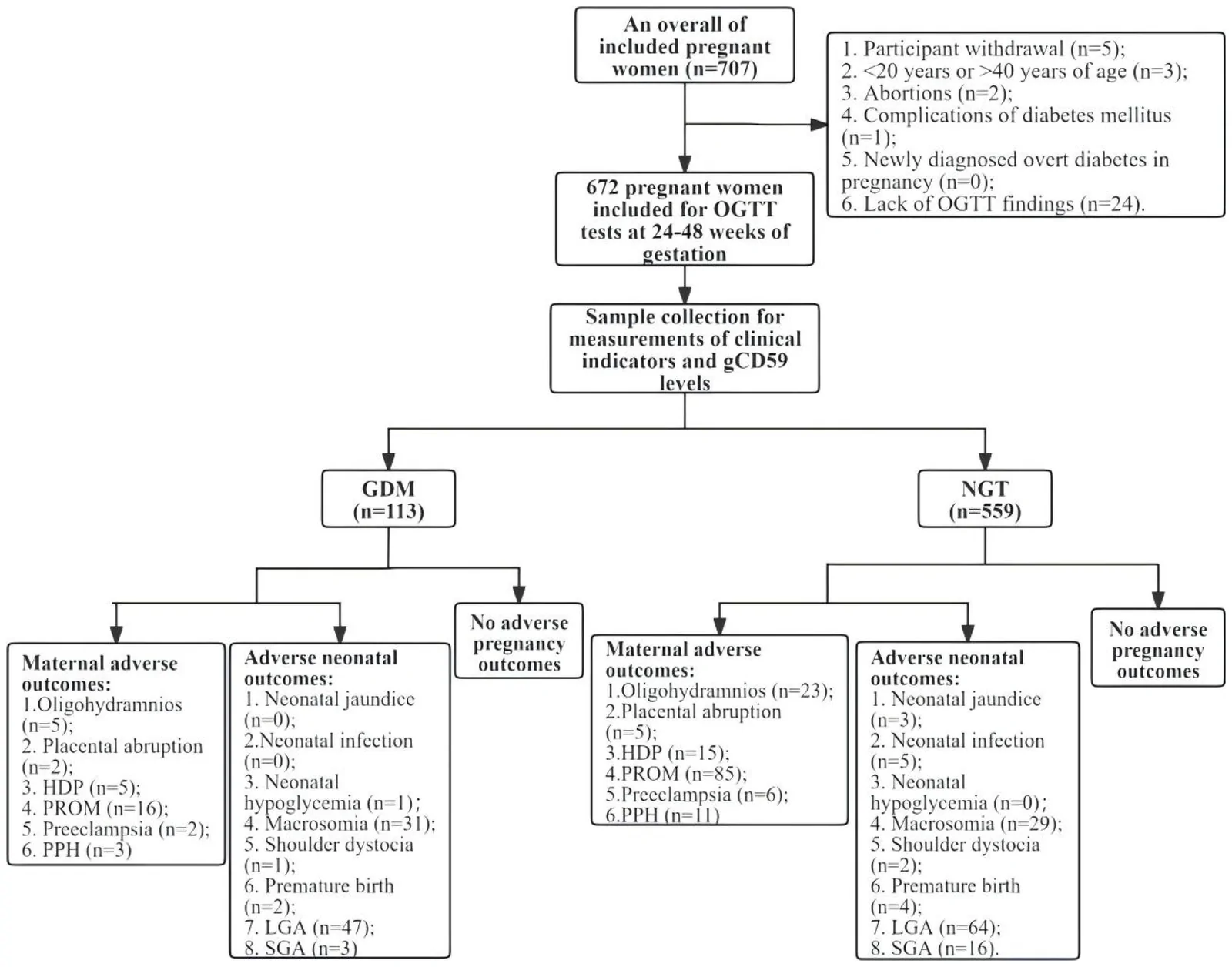
Flowchart. GDM, gestational diabetes mellitus; NGT, normal glucose tolerance; OGTT, oral glucose tolerance test; gCD59, glycated CD59.

### 2.2 Diagnosis of GDM

GDM was diagnosed via an oral glucose tolerance test (OGTT) at 24–28 weeks of gestation. In summary, a standard OGTT should be performed after overnight fasting (8–14 hours) by giving 75 g anhydrous glucose in 250–300 ml water. Cubital venous blood samples (2 mL) were harvested for the measurement of blood glucose levels during fasting, 1-h, and 2-h intervals using the serum glucose oxidase method. The diagnosis of GDM was confirmed on the basis of any one of the following thresholds, as outlined in the WHO Guidelines for Diagnosis and Management of Hyperglycemia in Pregnancy: fasting plasma glucose (FPG) ≥5.1 mmol/L, 1-h plasma glucose (PG) ≥10.0 mmol/L, and 2-h PG ≥8.5 mmol/L (9).

### 2.3 Measurements

#### 2.3.1 Demographics

Electronic medical records for pregnancy on the Smart Maternal and Child Health Service System (Shanghai Zhending Health Technology Co., Ltd., China) were established during the first visit in the obstetrics clinic, including age, sampling date, gravidity, parity, height, weight, and body mass index (BMI). The gestational age was estimated via a color Doppler ultrasound.

#### 2.3.2 Clinical indicators

Peripheral venous blood samples after an 8-h fasting were harvested in the morning from the cubital vein for measurements of clinical indicators, including hemoglobin (Hb), ferritin (FER), alanine aminotransferase (ALT), aspartate aminotransferase (AST), blood urea nitrogen (BUN), creatinine (Cr), uric acid (UA), triglyceride (TG), total cholesterol (TC), thyroid peroxidase antibody (TPOAb), free thyroxine (FT4), thyroid stimulating hormone (TSH), etc.

#### 2.3.3 gCD59 levels

Peripheral venous blood samples (5 mL) were harvested from participants after an interval of 8–14 h of fasting at the first visit and 24–28 wk of pregnancy, placed in EDTA tubes, and centrifuged at 3,000×g for 10 min at room temperature. The upper layer of 1 mL of separated plasma was transferred to a new tube, and prepared into two 500-µL samples for storage at -80 °C. As previously reported by Ghosh et al. (10), gCD59 levels were measured via enzyme-linked immunosorbent assay (ELISA) utilized in the preceding research (7). All samples were labeled and tested in the Central Laboratory of the Affiliated Suqian Hospital of Xuzhou Medical University. Investigators were blinded to the information about pregnant women and their blood glucose levels.

#### 2.3.4 Adverse pregnancy outcomes

All pregnant women were followed up until they were discharged after delivery, including gestational age at delivery, mode of delivery, 5-min Apgar score, neonatal gender, etc. Adverse pregnancy outcomes were categorized into maternal and neonatal. Maternal adverse outcomes consisted of oligohydramnios, hypertensive disorders of pregnancy (HDP), placental abruption, premature rupture of membranes (PROM), preeclampsia, and postpartum hemorrhage (PPH). Adverse neonatal outcomes included shoulder dystocia, premature birth, neonatal jaundice, Neonatal hypoglycemia, neonatal infection, macrosomia, large for gestational age (LGA), and small for gestational age (SGA).

Briefly, oligohydramnios was defined as an insufficient amniotic fluid <5 cm or the maximum vertical pocket <2 cm. HDP was confirmed by transient hypertension (systolic blood pressure [SBP] ≥140 mmHg or diastolic blood pressure [DBP] ≥90 mmHg), edema, proteinuria, and other symptoms in pregnant women after 20 wk of gestation, and normalized after delivery. Placental abruption was a severe condition after 20 wk of pregnancy when the placenta partly or completely detached from the inner wall of the uterus before delivery. PROM was a rupture of the amniotic sac at preterm (<37 weeks of gestation). Preeclampsia was diagnosed by the new-onset hypertension (SBP ≥140 mmHg or DBP ≥90 mmHg) and proteinuria (>300 mg/d) after 20 wk of gestation. PPH was characterized by excessive blood loss in vaginal delivery (>500 mL) or cesarean section (>1,000 mL) within 24 h. Shoulder dystocia was a complication of vaginal delivery caused by one or both shoulders of the fetus lodged against the pelvic bones of the pregnant woman. Premature birth was defined as neonates born alive before 37 wk of pregnancy. Neonatal jaundice is defined as meeting any one of the following criteria: 1) Onset of jaundice within 24 hours after birth. 2) The rate of increase in total serum bilirubin (TSB) exceeds 8.5μmol/(L·h) or 85μmol/L per day. 3) TSB level >221μmol/L in term infants and >257μmol/L in preterm infants. 4) Prolonged duration of jaundice: lasting more than 2 weeks in term infants and more than 4 weeks in preterm infants. 5) Recurrence of jaundice after initial resolution. 6) Conjugated bilirubin level >34μmol/L. Neonatal hypoglycemia is defined as a venous blood glucose concentration less than 2.2 mmol/L at any time after birth. Neonatal infection refers to infectious diseases in neonates caused by invasion of pathogens such as bacteria and viruses during the prenatal, intrapartum or postpartum periods. A newborn weighing more than 4000 g was diagnosed with macrosomia. LGA referred to a condition in which newborns weigh above the 90^th^ percentile for their gestational age, and SGA referred to a condition in which newborns weigh below the 10^th^ percentile for their gestational age.

Based on the established epidemiological evidence linking GDM with fetal overgrowth, macrosomia and LGA were pre-specified as the primary endpoints of this study. All other maternal outcomes (e.g., oligohydramnios, HDP, placental abruption, PROM, preeclampsia, PPH) and neonatal outcomes (e.g., shoulder dystocia, premature birth, neonatal jaundice, neonatal hypoglycemia, neonatal infection, SGA) were defined as secondary endpoints for exploratory analyses.

### 2.4 Statistical analysis

Statistical analysis was conducted using SPSS 26.0. Following the normality test, measurement data within a normal distribution were expressed as mean ± standard deviation ( $\bar{x}$ ± s), and subjected to between-group comparison by the Student’s t test. Those in a skewed distribution were expressed as medians and quartiles (M [P25, P75]), and analyzed by the Mann-Whitney U test. Enumeration data were presented as n (%), and subjected to between-group comparison using the Chi-square test or Fisher’s exact test. Risk factors for macrosomia and LGA were identified using univariate and binary logistic regression models. Multicollinearity among independent variables was assessed using the variance inflation factor (VIF), with VIF < 5 considered acceptable. All logistic regression models were performed on the entire cohort (N = 672), with GDM status (yes/no) included as a covariate in the multivariable models to adjust for its confounding effect. With the total cohort (N = 672), the number of events was 60 for macrosomia and 111 for LGA, providing an events-per-variable (EPV) ratio of approximately 6.7 and >12, respectively, which is acceptable for exploratory analyses. The potential of gCD59 in predicting macrosomia and LGA newborns delivered by mothers with GDM was examined by plotting the receiver operating characteristic (ROC) curves, with the estimations of 95% confidence interval (CI) and area under the curve (AUC). *P* < 0.05 suggested a significant difference. For secondary outcomes, *P*-values were adjusted using the Bonferroni correction to control for multiple testing.

## 3 Results

### 3.1 Clinical characteristics of pregnant women

To provide clinical context for the subsequent predictive models, we first compared baseline characteristics between the GDM and NGT groups. A total of 707 pregnant women who visited the obstetrics clinic for the first time were prospectively included in our cohort study. After excluding 5 cases of participant withdrawal, 3 cases younger than 20 years or older than 40 years, 2 cases of abortions, 1 case of newly diagnosed overt diabetes in pregnancy, and 24 cases without OGTT findings, 672 eligible pregnant women were finally retained and assigned into the GDM group (n=113, 16.8%) and normal glucose tolerance (NGT) group (n=559, 83.2%) based on OGTT results. The median level of gCD59 was significantly higher in the GDM group than the NGT group (1.49 signal producing units [SPU] vs. 0.87 SPU, *P* < 0.001). Compared to those in the NGT group, FPG, 1-h PG, 2-h PG, blood pressure, pre-pregnancy weight, and BMI were significantly higher in the GDM group (all *P* < 0.001). Pregnant women with GDM presented significantly higher TG levels in comparison to those with NGT (*P* < 0.05). Demographic and clinical characteristics of pregnant women with GDM versus NGT are detailed in **Table 1**.These descriptive comparisons serve to illustrate the background risk profile of the cohort and are not the primary analytical focus of this study. Collinearity diagnostics showed that the VIF values for all covariates ranged from 1.109 to 2.045, all well below the threshold of 5, indicating no significant multicollinearity among the independent variables. All subsequent logistic regression and ROC analyses were performed on the entire cohort (N = 672), with GDM status included as a covariate in all multivariable models.

**Table 1:** Comparison of baseline data between the Two Groups.

Baseline characteristics	GDM(n=113)	NGT(n=559)	*P* Value
FPG (mmol/L)	5.21 (4.93,5.46)	4.52 (4.26,4.73)	<0.001
1h glucose (mmol/L)	9.20 (7.21,10.31)	7.22 (6.32,8.16)	<0.001
2h glucose (mmol/L)	8.07 (6.63,8.99)	6.30 (5.64,7.07)	<0.001
Age (years)	30 (27,33)	30 (27,33)	0.40
Gravidity	2 (1,3)	2 (1,3)	0.12
Parity	2 (1,2)	1 (1,2)	0.15
Height (cm)	162 (160,165)	163 (160,165)	0.53
Weight (kg)	62.5 (57.25,70)	58 (52.5,65)	<0.001
BMI (kg/m^2^)	24.06 (22.04,24.07)	21.87 (20,21.88)	<0.001
SBP (mmHg)	112 (106,124)	110 (104,118)	<0.001
DBP (mmHg)	73 (68.25,80)	69 (65,74)	<0.001
Ferritin (ng/ml)	15.7 (8.7,26.3)	13.5 (7.8,23.9)	0.53
Hb (g/L)	119 (112,124)	118 (112,124)	0.68
BUN (mmol/L)	2.78 (2.5,3.46)	2.77 (2.31,3.28)	0.19
Cr (μmol/L)	45 (40.6,49)	45 (40.5,49)	0.68
UA (μmol/L)	245.7 (209.63,297.58)	240.1 (203.7,282.2)	0.33
TG (mmol/L)	1.79 (1.34,2.9)	1.56 (1,2.75)	0.01
TC (mmol/L)	4.65 (4.03,6.4)	5.18 (4.18,6.61)	0.13
ALB (g/L)	37.3 (35.35,41.35)	38.3 (35.7,41.8)	0.18
ALT (U/L)	12.35 (8.68,17.03)	12 (9,16.8)	0.76
AST (U/L)	15.65 (13.68,18.48)	16.1 (14,18.8)	0.45
TPOAB (IU/mL) (+)	9 (7.96%)	26 (4.65%)	0.15
FT4 (pmol/L)	12.65 (11.4,14.6)	12.6 (11.4,13.8)	0.72
TSH (μIU/mL)	1.94 (1.28,2.85)	1.83 (1.25,2.54)	0.15
gCD59 (SPU)	1.49 (0.90,2.33)	0.87 (0.39,1.62)	<0.001

GDM, gestational diabetes mellitus; NGT, normal glucose tolerance; FPG, fasting plasma glucose; BMI, body mass index; SBP, systolic blood pressure; DBP, diastolic blood pressure; Hb, hemoglobin; BUN, blood urea nitrogen; Cr, Creatinine; UA, uric acid; TG, triglyceride; TC, total cholesterol; ALB, albumin; ALT, alanine aminotransferase; AST, aspartate aminotransferase; TPOAB, Thyroid Peroxidase Antibody; FT4, free theroxine; TSH, thyroid stimulating hormone.

### 3.2 Adverse pregnancy outcomes of GDM

The incidence of adverse maternal outcomes was comparable between the GDM and NGT groups (*P*>0.05). The detailed adverse pregnancy outcomes are shown in **Table 2**. However, adverse neonatal outcomes were more frequently detected in newborns delivered by mothers with GDM than those with NGT. In comparison to the NGT group, the incidence of macrosomia (27.4% vs. 5.2%) and LGA (41.6% vs. 11.4%) was significantly higher in the GDM group (both *P* < 0.001).These findings confirm the established association between GDM and fetal overgrowth in our cohort, providing biological plausibility for our subsequent predictive analyses. After Bonferroni correction for multiple testing, none of the secondary maternal or neonatal outcomes showed statistically significant differences between the two groups, consistent with the uncorrected analyses.

**Table 2:** Comparison of Adverse Pregnancy Outcomes between the Two Groups.

Outcome	GDM (n=113)	NGT (n=559)	*P* value
Maternal adverse outcomes			
Oligohydramnios	5 (4.4%)	23 (4.1%)	0.88
HDP	5 (4.4%)	15 (2.7%)	0.5
Placental abruption	2 (1.8%)	5 (0.9%)	0.88
PROM	16 (14.2%)	85 (15.2%)	0.35
Preeclampsia	2 (1.8%)	6 (1.1%)	0.68
PPH	3 (2.7%)	11 (2.0%)	0.83
Adverse neonatal outcomes			
Shoulder dystocia	1 (0.9%)	2 (0.4%)	0.53
Premature birth	2 (1.8%)	4 (0.7%)	0.72
Neonatal jaundice	0 (0%)	3 (0.5%)	0.53
Neonatal infection	0 (0%)	5 (0.9%)	0.51
Neonatal hypoglycemia	1 (0.9%)	0 (0.0%)	0.68
Macrosomia	31 (27.4%)	29 (5.2%)	<0.001
LGA	47 (41.6%)	64 (11.4%)	<0.001
SGA	3 (2.7%)	16 (2.9%)	0.93

HDP, hypertensive disorders of pregnancy; PROM, Premature rupture of membranes; PPH, postpartum hemorrhage; LGA, large for gestational age; SGA, small for gestational age.

### 3.3 Risk factors for macrosomia and LGA

A binary logistic regression model adjusted for body weight, BMI, SBP, and DBP (Model 1a) was created with macrosomia as the dependent variable, demonstrating that gCD59 was a risk factor for macrosomia (OR = 3.492). Following additional adjustments for FBT, TG, TSH, ferritin and GDM status (Model 2a), it is found that gCD59 remained an independent risk factor for macrosomia. For every 1 SPU increase in gCD59, the risk of macrosomia increased by 3.15 times (**Table 3**).

**Table 3:** Logistic regression analysis of risk factors for macrosomia.

Variable	Model 1a		Model 2a	
	OR (*95%CI*)	*P* value	OR (*95%CI*)	*P* value
gCD59	3.492 (2.455-4.966)	<0.001	3.152 (1.972-5.037)	<0.001
Weight	0.986 (0.910-1.069)	0.735	0.983 (0.885-1.091)	0.748
BMI	1.207 (0.965-1.508)	0.099	1.213 (0.912-1.614)	0.185
SBP	1.027 (0.989-1.068)	0.169	1.033 (0.986-1.082)	0.177
DBP	0.963 (0.917-1.011)	0.125	0.975 (0.918-1.036)	0.417
FBG			1.794 (0.682-4.723)	0.237
TG			0.800 (0.555-1.154)	0.233
TSH			0.931 (0.666-1.302)	0.676
Ferritin			0.996 (0.973-1.020)	0.747
GDM status			2.297 (0.756-6.982)	0.142

Model 1, Adjusted for weight, BMI, SBP, and DBP; Model 2, On the basis of Model 1, further adjust for FBG, TG, TSH ferritin and GDM status.

In addition, a binary logistic regression model controlling for confounders of body weight, BMI, SBP, and DBP (Model 1b) was created to predict LGA (the dependent variable), revealing that gCD59 (OR = 2.406) and weight (OR = 1.078) were independent risk factors for LGA. In Model 2b with adjustments for body weight, BMI, SBP, DBP, FBG, TG, TSH, ferritin and GDM status, it is found that gCD59 (*P* < 0.001), and TSH (*P* < 0.05) were identified as independent predictors of LGA. Specifically, each 1 SPU increase in gCD59 was associated with an approximately 2.33-fold increased risk of LGA, while each 1 μIU/mL increase in TSH was associated with an approximately 30.9% reduced risk. (**Table 4**).

**Table 4:** Logistic Regression Analysis of Risk Factors for LGA.

Variable	Model 1b		Model 2b	
	OR *(95%CI)*	*P* value	OR *(95%CI)*	*P* value
gCD59	2.406 (1.836-3.152)	<0.001	2.329 (1.624-3.338)	<0.001
Weight	1.078 (1.004-1.157)	0.039	1.062 (0.971-1.162)	0.189
BMI	0.931 (0.765-1.134)	0.477	0.943 (0.735-1.209)	0.643
SBP	0.987 (0.957-1.017)	0.387	1.007 (0.970-1.045)	0.723
DBP	1.006 (0.968-1.045)	0.758	0.998 (0.952-1.045)	0.917
FBG			1.722 (0.757-3.915)	0.192
TG			1.031 (0.858-1.240)	0.743
TSH			0.691 (0.502-0.951)	0.023
Ferritin			1.016 (0.997-1.034)	0.100
GDM status			2.109 (0.828-5.305)	0.113

Model 1, Adjusted for weight, BMI, SBP, and DBP; Model 2, On the basis of Model 1, further adjust for FBG, TG, TSH ferritin and GDM status.

### 3.4 Incremental predictive value of gCD59 for macrosomia and LGA

We further evaluated the incremental predictive value of gCD59 beyond traditional risk factors. Two multivariable logistic regression models were constructed: Model A (traditional risk factors: BMI, gestational weight gain, FBG, and GDM status) and Model B (Model A + gCD59). The predicted probabilities from both models were used to plot ROC curves, and the AUCs were compared using the DeLong test. For macrosomia, Model A achieved an AUC of 0.770 (95% CI: 0.679-0.833), while Model B achieved an AUC of 0.864 (95% CI: 0.823–0.904), with a DeLong test *P* = 0.046, indicating that the addition of gCD59 significantly improved predictive performance (**Figure 2**). For LGA, Model A achieved an AUC of 0.723 (95% CI: 0.663–0.782), while Model B achieved an AUC of 0.790 (95% CI: 0.737–0.843), with a DeLong test *P* = 0.103, indicating a favorable trend that did not reach statistical significance (**Figure 3**). In addition, supplementary ROC analyses using gCD59 alone were performed for reference (**Supplementary Figure S1** for macrosomia and S2 for LGA), with the results presented in the Discussion.

**Figure 2:**
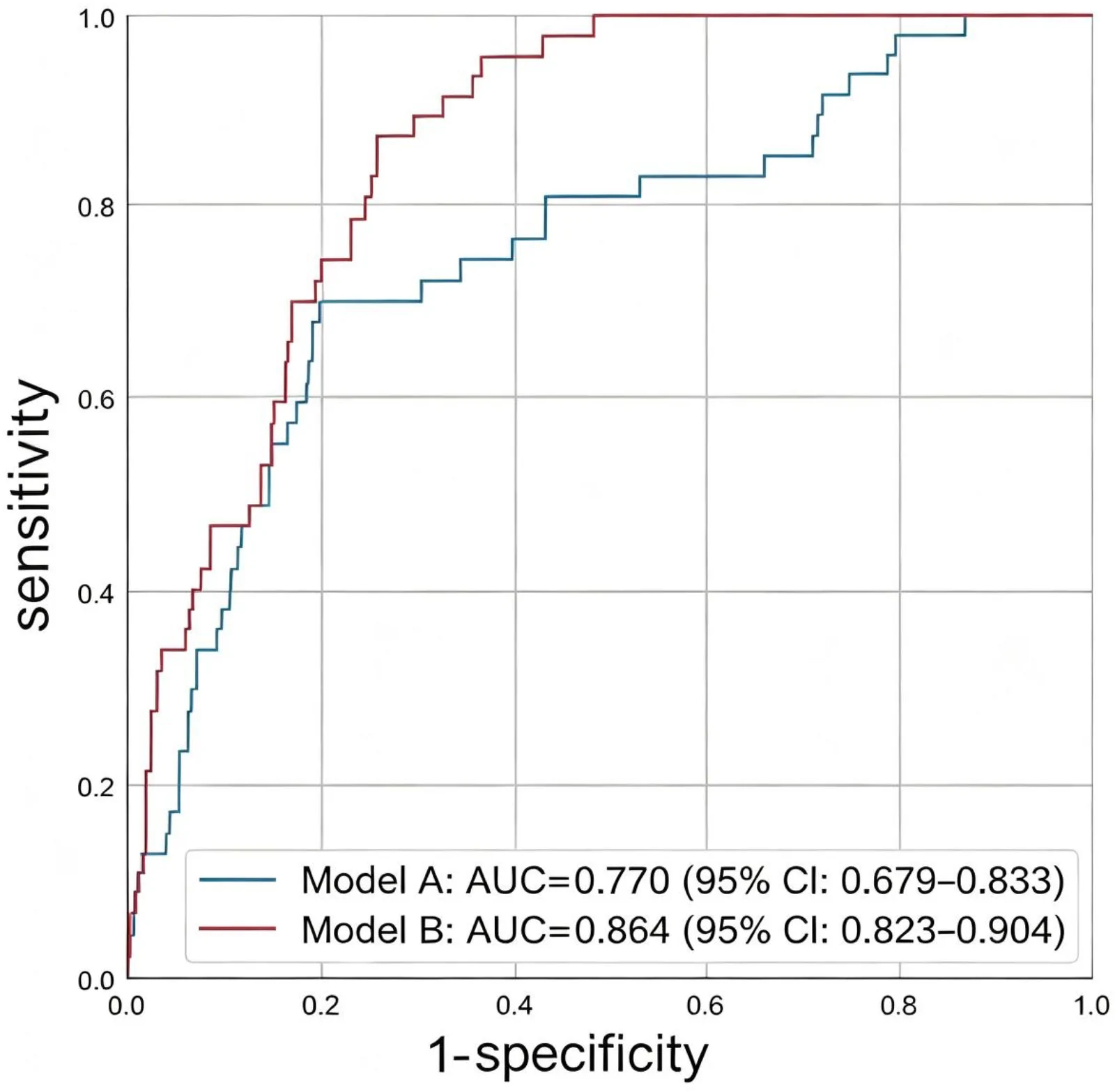
Receiver operating characteristic (ROC) curves for predicting macrosomia. The blue line represents Model A (traditional risk factors: BMI, gestational weight gain, FBG, and GDM status). The red line represents Model B (Model A + gCD59).

**Figure 3:**
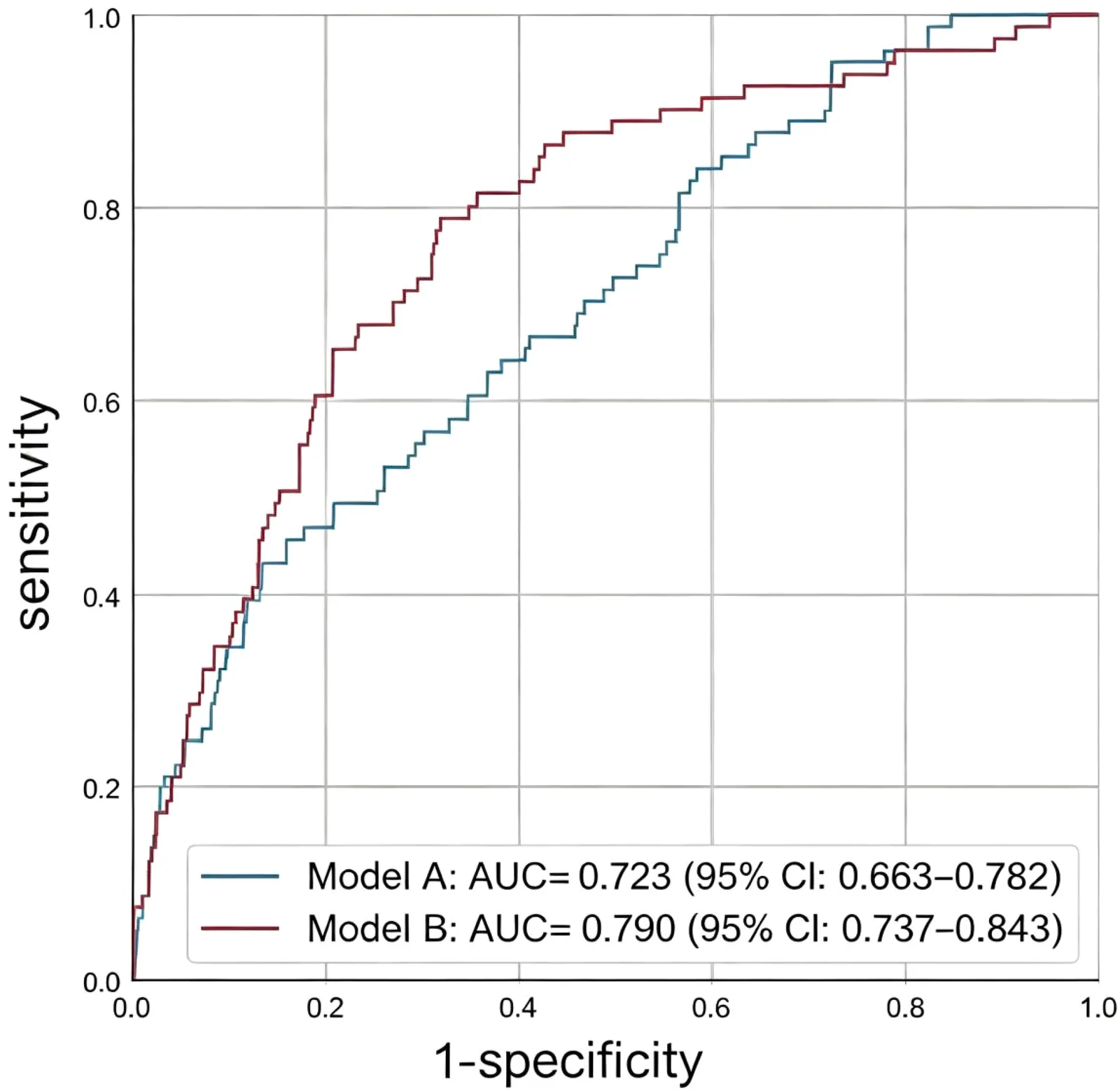
Receiver operating characteristic (ROC) curves for predicting large for gestational age (LGA). The blue line represents Model A (traditional risk factors: BMI, gestational weight gain, FBG, and GDM status). The red line represents Model B (Model A + gCD59).

## 4 Discussion

Pregnant women with GDM are confronted with a risk of adverse pregnancy outcomes. Consequently, an early recognition of the high-risk population of GDM is of clinical significance to improve pregnancy outcomes. Our previous work has validated that gCD59, a potential biomarker for GDM (7), has a close association with adverse pregnancy outcomes (11, 12). In this prospective study involving a cohort of Chinese pregnant women, we demonstrated that macrosomia and LGA were the most common adverse pregnancy outcomes of GDM. More importantly, gCD59 was found as an independent risk factor for macrosomia and LGA. In predictive modeling, the addition of gCD59 to traditional risk factors significantly improved the AUC for macrosomia from 0.770 to 0.864 (DeLong *P* = 0.046), while for LGA the AUC improved from 0.723 to 0.790 (DeLong *P* = 0.103), suggesting a potential role for gCD59 as a supplementary biomarker, particularly for macrosomia risk stratification.

The prevalence of GDM was 16.8% in our cohort of 672 included pregnant women, which is consistent with the epidemiological finding of GDM in China. GDM, an established risk for adverse pregnancy outcomes, significantly increased the prevalences of macrosomia and LGA in the GDM group than the NGT group. Generally, an integration of maternal, placental, and fetal factors jointly determines the pregnancy outcomes of GDM. Maternal metabolic disorders impair the normal placental function and hamper the nutritional supply to fetuses, eventually resulting in fetal hyperinsulinemia (13). Previous evidence has demonstrated that age and pre-pregnancy BMI of mothers are metabolic risk factors for adverse pregnancy outcomes (14). Disruptions into both the placental function and the maternal glucose-insulin axis may produce poor fetal outcomes, as evidenced by a close association of relevant serum markers with adverse birth outcomes (15, 16).

In the present study, logistic regression proved that gCD59 was an independent risk factor for macrosomia and LGA after adjusting for baseline metabolic indicators and laboratory indicators. For every 1 SPU increase in gCD59, the risk of macrosomia and LGA increased by 3.15 and 2.33 times, respectively, indicating that gCD59 may contribute to the risk of adverse pregnancy outcomes. A graded relationship between gCD59 levels and the prevalence of macrosomia and LGA was also observed in descriptive analyses (**Supplementary Figure 1**), further supporting the regression findings. In our previous study, we have validated the diagnostic potential of plasma gCD59 in GDM. At present, the association of gCD59 with adverse pregnancy outcomes has not been extensively analyzed. Ghosh et al. (17) measured gCD59 during the second trimester (24–28 weeks) and, as a secondary analysis, reported a positive correlation with the incidence of LGA. Ma et al. (18), in a cohort of high-risk overweight/obese women enrolled before 20 weeks of gestation, demonstrated that first-trimester gCD59 predicted LGA newborns, without a significant association with macrosomia. Here, we proved that gCD59 was an independent risk factor for macrosomia and LGA in Chinese pregnant women. CD59, a complement regulatory protein, normally protects against MAC deposition. Glycation inactivation of CD59, however, forms gCD59 to accelerate local MAC accumulation and activate the intracellular MAC signaling (19), thereafter altering placental structure and function, and ultimately raising the risk of adverse pregnancy outcomes during pregnancy (20).

To date, the potential of gCD59 in predicting adverse pregnancy outcomes has not been broadly analyzed. Bogdanet et al. (21) have reported that the AUC values of gCD59 in predicting macrosomia and LGA in a cohort of 378 pregnant women are 0.63 and 0.66, respectively. In our supplementary analyses using gCD59 alone (**Supplementary Figures S1**, **S2**), the AUC values for predicting macrosomia and LGA were 0.775 and 0.705, respectively, which are higher than those reported by Bogdanet et al. The smaller AUC of gCD59 reported by Bogdanet et al. (21) might be attributed to a better glucose status in the second trimester, potentially benefiting from early recognition and interventions of GDM by monitoring gCD59 and blood glucose levels in both the first and second trimesters. Bogdanet et al. (21) have further validated a superior ability of first-trimester gCD59 than the second-trimester value in predicting macrosomia and LGA. A relevant study has consistently shown the stronger ability of first-trimester gCD59 in predicting GDM (6), highlighting the clinical significance of diagnosing abnormal glucose metabolism in early pregnancy. The potential of first-trimester gCD59 in predicting adverse pregnancy outcomes among Chinese pregnant women requires further investigation.

The differential predictive performance of gCD59 for macrosomia versus LGA warrants further discussion. Macrosomia, defined as an absolute birth weight exceeding 4000 g, represents a more extreme metabolic phenotype than LGA, which is defined relative to gestational age percentiles. The stronger and statistically significant incremental value observed for macrosomia (DeLong *P* = 0.046) is biologically plausible, as gCD59 is a marker of cumulative glycemic exposure, and more severe fetal overgrowth is likely driven by greater maternal hyperglycemia. The non-significant result for LGA (DeLong *P* = 0.103) may be influenced by the heterogeneity inherent in percentile-based definitions across different gestational age strata, as well as the relatively modest sample size. Nevertheless, the consistent trend toward improved AUC for both outcomes supports the potential clinical utility of gCD59 as an adjunctive risk stratification tool, particularly for identifying pregnancies at highest risk of excessive fetal growth.

Beyond its statistical association, the clinical utility of gCD59 as a serum biomarker warrants consideration. Unlike OGTT, which requires oral glucose loading and multiple blood draws, gCD59 can be measured from a single fasting blood sample, offering greater patient convenience and potentially earlier detection of glycemic abnormalities. Furthermore, its measurement is not limited to the 24–28 week window, making it theoretically applicable for first-trimester risk stratification. However, the additional cost of gCD59 assay and its incremental value over existing screening tools must be carefully evaluated in cost-effectiveness studies before routine clinical implementation can be recommended.

Limitations in the present study should be noted. First, the small sample size may potentially lead to biases. Multi-center studies with a large sample size are essential to validate our findings. Second, although gCD59 showed significant incremental predictive value for macrosomia, the improvement for LGA did not reach statistical significance. This may be due to the heterogeneity of LGA definitions across gestational age strata and the relatively limited sample size, which should be addressed in future larger-scale studies. Third, adverse pregnancy outcomes may arise from the cooperation of maternal, placental, and fetal factors. Single use of gCD59 may be less reliable in predicting adverse pregnancy outcomes. A combination of gCD59 with multi-dimensional biomarkers is expected to more accurately predict adverse pregnancy outcomes.

Taken together, macrosomia and LGA were the leading adverse pregnancy outcomes among Chinese pregnant women with GDM. gCD59 appeared as an independent risk factor and might serve as a novel biomarker for predicting macrosomia and LGA. In the future, the potential of gCD59 in predicting adverse pregnancy outcomes among distinct populations and across stages of pregnancy should be further explored.

## Data Availability

The original contributions presented in the study are included in the article/**Supplementary Material**, further inquiries can be directed to the corresponding authors.
