# Supplementary material for: The predictive value of glycated CD59 for fetal overgrowth in second-trimester pregnant women
Source: Front Endocrinol (Lausanne). 2026 Oct 1;17:1924289. doi: 10.3389/fendo.2026.1924289 (PMC13668522; doi:10.3389/fendo.2026.1924289)
Supplement: Supplementary file 1 [file SupplementaryFile1.docx]

Supplementary Figure S1 and S2:

Figure S1: ROC curve of gCD59 for predicting macrosomia

Figure S2: ROC curve of gCD59 for predicting LGA

Supplementary Figure S3 and S4:

Figure S3: ROC curve of gCD59 for predicting macrosomia within the GDM subgroup

Figure S4: ROC curve of gCD59 for predicting LGA within the GDM subgroup

Supplementary Figure 1：This figure was relocated from the main text to the supplementary materials following reviewer suggestion.

Supplementary Figure 1: Prevalence of macrosomia and LGA by quartiles of gCD59
